# Heart Rate Recovery in Patients With Hypertrophic Cardiomyopathy

**DOI:** 10.1016/j.amjcard.2013.11.062

**Published:** 2014-03-15

**Authors:** Vimal Patel, Christopher H. Critoph, Malcolm C. Finlay, Bryan Mist, Pier D. Lambiase, Perry M. Elliott

**Affiliations:** Inherited Cardiac Diseases Unit, The Heart Hospital, University College London, London, United Kingdom

## Abstract

Recovery in heart rate (HR) after exercise is a measure of autonomic function and a prognostic indicator in cardiovascular disease. The aim of this study was to characterize heart rate recovery (HRR) and to determine its relation to cardiac function and morphology in patients with hypertrophic cardiomyopathy (HC). We studied 18 healthy volunteers and 41 individuals with HC. All patients underwent clinical assessment and transthoracic echocardiography. Continuous beat-by-beat assessment of HR was obtained during and after cardiopulmonary exercise testing using finger plethysmography. HRR and power spectral densities were calculated on 3 minutes of continuous RR recordings. Absolute HRR was lower in patients than that in controls at 1, 2, and 3 minutes (25.7 ± 8.4 vs 35.3 ± 11.0 beats/min, p <0.001; 36.8 ± 9.4 vs 53.6 ± 13.2 beats/min, p <0.001; 41.2 ± 12.2 vs 62.1 ± 14.5 beats/min, p <0.001, respectively). HRR remained lower in patients at 2 and 3 minutes after normalization to peak HR. After normalization to the difference in HR between peak exercise and rest, HRR was significantly impaired in individuals with obstructive HC at 3 minutes compared with controls. HR at 3 minutes correlated with peak left ventricular outflow tract gradient (B 0.154 beats/min/mm Hg, confidence interval 0.010 to 0.299, p = 0.037) and remained a significant predictor of HRR after multivariable analysis. Spectral analysis showed a trend toward an increased low-frequency to high-frequency ratio in patients (p = 0.08) suggesting sympathetic predominance. In conclusion, HRR is impaired in HC and correlates with the severity of left ventricular outflow tract gradient. Prospective studies of the prognostic implications of impaired HRR in HC are warranted.

Heart rate recovery (HRR) after exercise is a product of vagal reactivation and sympathetic withdrawal.^1^ Impairment of HRR after cardiopulmonary exercise testing is associated with increased cardiovascular and all-cause mortalities.^2–7^ As patients with hypertrophic cardiomyopathy (HC) have evidence for altered autonomic function^8–13^ we hypothesized that HRR responses are abnormal in HC. The aim of this study was to characterize HRR responses and to determine their relation to cardiac morphology and conventional risk factors for sudden cardiac death.

## Methods

The study cohort comprised 47 consecutive patients attending a dedicated cardiomyopathy clinic at the Heart Hospital, University College London Hospitals, London, UK. The study complies with the principles of Declaration of Helsinki and National Health Service research governance arrangements.

Twenty healthy volunteers with no medical conditions or family history of inherited heart disease were recruited as controls. All participants were in sinus rhythm with no concurrent diagnosis of anemia. One patient had type 1 diabetes mellitus. Two healthy controls were considered to be athletically trained based on their reported level of activity and exercise capacity (peak oxygen consumption >170% predicted) and were excluded from subsequent analysis. Six patients performed a submaximal exercise test (respiratory quotient <1.05) and were excluded.

Before exercise, all patients underwent clinical assessment using 2-dimensional transthoracic and Doppler echocardiography, 12-lead electrocardiography, and lung spirometry. All patients fulfilled current diagnostic criteria for HC based on 2-dimensional echocardiography (maximal wall thickness ≥15 mm unexplained by abnormal loading conditions).^14^

Measurement of left ventricular maximal wall thickness (MWT), left atrial dimension, and left ventricular dimensions at end-systole and end-diastole was performed using 2-dimensional echocardiography in accordance with previously published methods.^15^ Ejection fraction was calculated using Simpson's biplane method. Left ventricular outflow tract obstruction (LVOTO) was measured using continuous wave Doppler; LVOTO was defined as a resting left ventricular outflow gradient of ≥30 or ≥50 mm Hg on physiological provocation (Valsalva or exercise).

New York Heart Association functional class and the following risk factors for sudden cardiac death were documented: nonsustained ventricular tachycardia (NSVT; 3 or more consecutive ventricular extrasystoles at a rate of ≥120 beats/min) on ambulatory monitoring, unexplained syncope, MWT ≥30 mm, family history of sudden cardiac death, and an abnormal blood pressure response (ABPR) to exercise (systolic blood pressure <20 mm Hg).^14^

Beta blockers and calcium channel antagonists were withheld for at least 24 hours before exercise. Two patients with nonobstructive HC in the final study cohort were on amiodarone therapy. Five patients were receiving therapy with angiotensin-converting enzyme inhibitors, and 4 patients were on diuretics. Only 1 patient with nonobstructive HC received antihypertensive therapy (angiotensin-converting enzyme inhibitor, loop diuretic, and aldosterone antagonist) on the day of the assessment. Exercise was performed in an upright position using a bicycle ergometer (ergoselect 200P; Ergoline, Germany) and a ramp protocol in a quiet air-conditioned room with an average temperature of 21°C and full resuscitation facilities. Before the test, the exercise procedure was explained, and all subjects were given the opportunity to become familiar with the technique at zero workload. Patients were instructed to pedal at a speed of 60 to 70 revolutions per minute and were encouraged to exercise to maximal capacity. Breath-by-breath gas exchange analysis was performed using a dedicated Sensormedics metabolic cart (V Max ENCORE 229 Console; Sensormedics, Viasys Healthcare, United Kingdom). Respiratory gases were sampled continuously from a mouthpiece and analyzed using an electrochemical cell oxygen analyzer and nondispersive infrared thermopile carbon dioxide analyzer. Peak oxygen consumption (VO_2_), workload (W), and respiratory exchange ratio were recorded.

Continuous beat-by-beat assessment of heart rate was obtained using finger plethysmography (Finometer; Finapres Medical Systems, The Netherlands). This was attached to the index finger of the left hand and maintained in neutral position using a specially made splint. Output was fed into a PC running Beatscope Easy software (Finapres Medical Systems, the Netherlands). At the point of exhaustion, the workload was returned to zero, and individuals were requested to remain static in an upright position for 3 minutes. HR in recovery was analyzed as absolute HR, HR normalized to the peak HR, and HR normalized to the change in HR from baseline to peak exercise. HR at 1, 2, and 3 minutes during recovery was obtained by calculating a mean HR from 10 consecutive beats at each respective time point. Analysis was performed on the absolute HR reduction and also on the percentage HRR standardized to peak HR and change in heart rate between baseline and peak exercise.

Power spectral densities were calculated on 3 minutes of continuous RR recordings obtained during recovery using Welch, autoregressive (Levinson-Durbin algorithm), and Lomb Scargle methods. Although absolute numbers varied, trends and group differences were similar between methods. Data from the Welch method are presented. Low-frequency and high-frequency raw powers (ms^2^) were normalized by total power after removal of the very low frequency component and were expressed as normalized units (n.u). Analysis was performed in heart rate variability (HRV) analysis software package, 1.0.1, using MATLAB 7.12 (MathWorks, Massachusetts).

After confirmation of normality, continuous variables are expressed as mean and SD, and categorical variables are shown as frequencies and percentages. Frequencies were compared using Fisher's exact test. Comparisons between groups were performed using independent *t* test and analysis of variance test with post hoc (Bonferroni) analyses to identify intergroup differences. Data that were not normally distributed are expressed as median and interquartile range and analyzed using nonparametric testing.

We sought to determine the influence of demographic and disease-specific variables on HRR using 2 models. As post hoc analysis identified significant differences between controls and patients, the first model used multivariable analysis with backward elimination and pooled patient and control data to determine the relation of a diagnosis of HC, gender, age, peak HR achieved, body mass index, and oxygen consumption at peak exercise to HR at 1, 2, and 3 minutes after exercise. To assess the influence of disease-specific variables, a second model was constructed in the patient group alone. A univariable analysis of 5 clinical surrogates for disease severity—lateral E/E′, left atrial area, ejection fraction, MWT, and left ventricular outflow gradient—and their relation to HR at 1, 2, and 3 minutes after exercise was performed using linear regression. Significant predictors were then added to an analysis that included significant (p <0.05) independent predictors of HRR identified from the first model. Statistical comparisons were carried out using IBM SPSS Statistics, version 20.0 and R 2.14 (*CRAN.R-project.org*). A p value of <0.05 was considered significant in all analyses.

## Results

The final study cohort consisted of 18 controls and 41 patients with HC. Characteristics of the study groups are listed in Table 1. The control group had equal numbers of men and women, although the HC group was predominantly men. Compared with the controls, patients were older with higher body mass index and lower HR, oxygen consumption, and workload at peak exercise. Resting HR and mean arterial pressure were similar in both groups.

**Figure 1:**
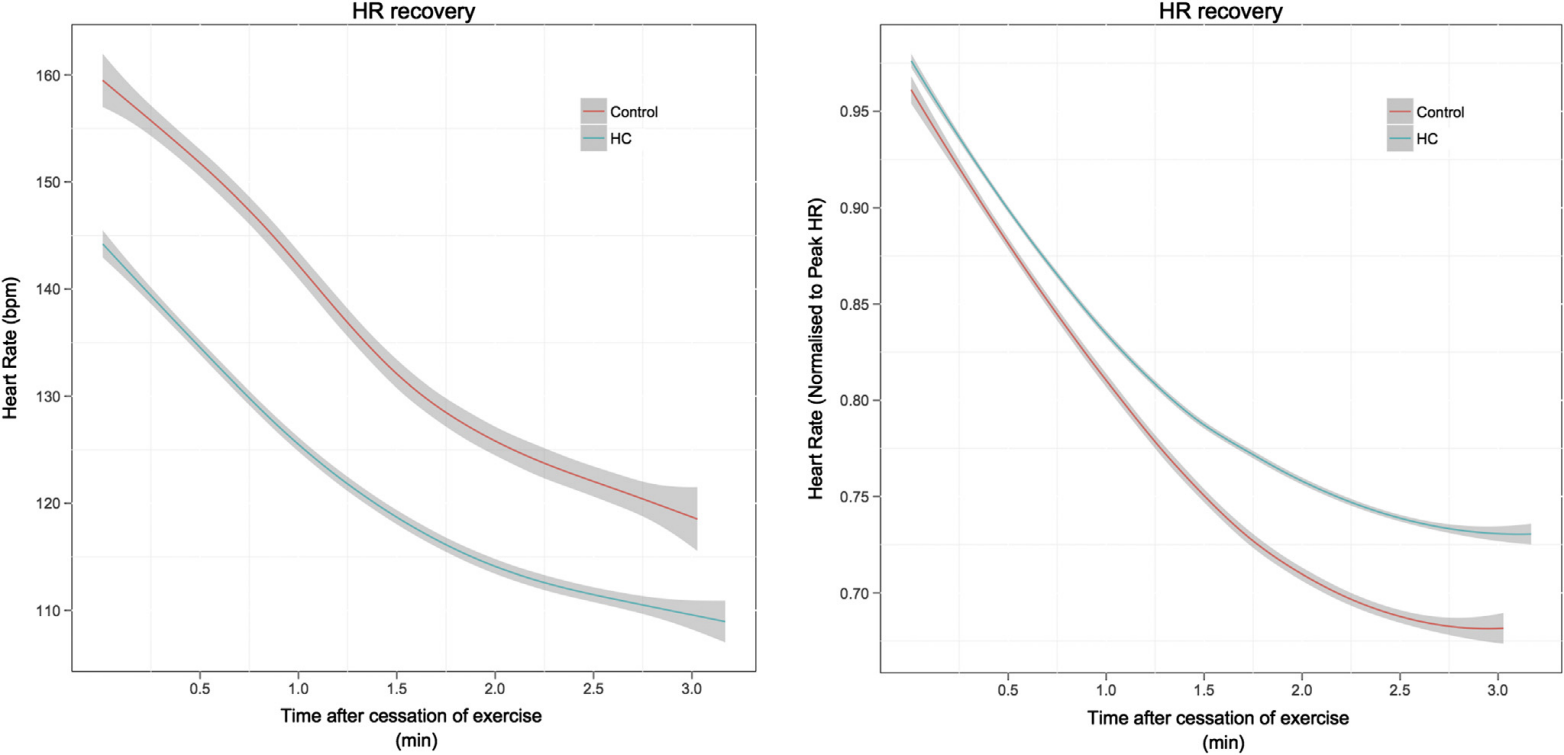
*(A)* Absolute HRR after symptom limited cardiopulmonary exercise between control and HC groups. The change in mean absolute heart rate with time after cessation of exercise is shown, with 95% CIs calculated by the Loess function. *(B)* HRR normalized to peak HR after symptom limited cardiopulmonary exercise between control and HC groups. The change in mean normalized heart rate with time after cessation of exercise is shown, with 95% CIs calculated by the Loess function.

The absolute recovery in HR and HRR normalized to peak HR is shown in Figure 1. At 1 minute, the absolute reduction in HR was lower in the HC group (25.7 ± 8.4 vs 35.3 ± 11.0 beats/min, p <0.001). When normalized to peak HR, there was a trend toward a slower recovery in HR at 1 minute in the HC group (17.2 ± 5.2% vs 20.4 ± 6.8%, p = 0.052). When normalized to the change in HR from rest to peak exercise, there was no significant difference between the groups at 1 minute (36.3 ± 11.9% vs 37.7 ± 13.5%, p = 0.680). At 2 minutes, the absolute reduction in HR was lower in HC (36.8 ± 9.4 vs 53.6 ± 13.2 beats/min, p <0.001). This remained significant after normalizing to peak HR (24.5 ± 5.2% vs 30.7 ± 7.8%, p = 0.01). When normalized to the change in HR from rest and peak exercise, there was no significant difference between the groups at 2 minutes (51.7 ± 10.9% vs 56.6 ± 13.9%, p = 0.148). At 3 minutes, the absolute reduction in HR (41.2 ± 12.2 vs 62.1 ± 14.5 beats/min, p <0.001) and the reduction indexed to peak HR (27.3 ± 6.4% vs 35.5 ± 8.6%, p <0.001) were smaller in HC group. When normalized to the change in HR from rest to peak exercise, there was a trend toward a lower HRR in the HC group (57.76 ± 13.48% vs 65.67 ± 15.80%, p = 0.054).

The characteristics of the cohort dichotomized according to the presence or absence of a resting left ventricular outflow tract gradient are listed in Table 1. Resting HR was similar in both disease subgroups. Peak HR was higher in controls compared with the nonobstructive and obstructive groups. Peak oxygen capacity was higher in controls compared with the nonobstructive and obstructive patients.

**Figure 2:**
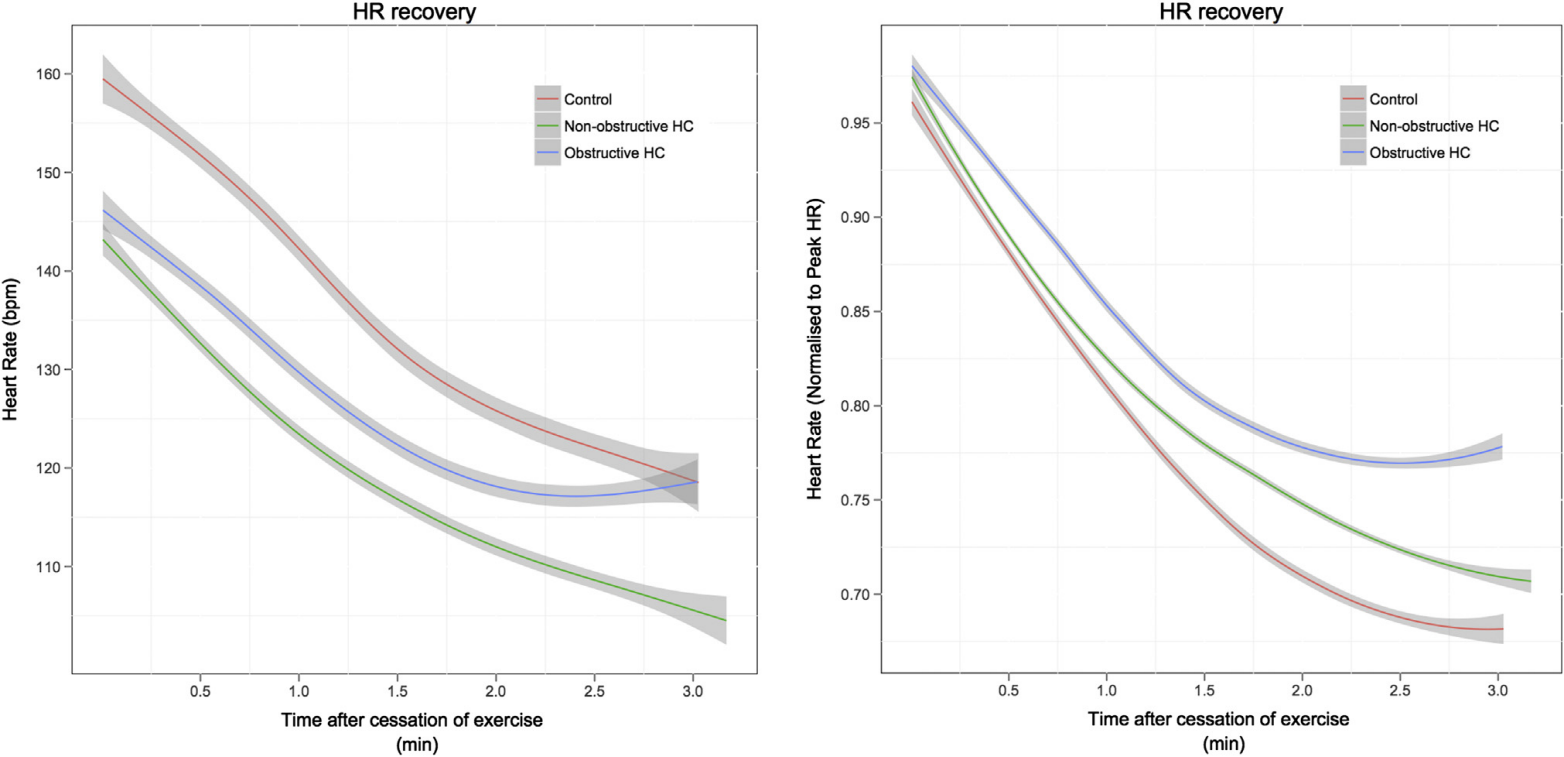
*(A)* Absolute HRR after symptom limited cardiopulmonary exercise among control, nonobstructive, and obstructive groups. The change in mean absolute heart rate with time after cessation of exercise is shown, with 95% CIs calculated by the Loess function. *(B)* HRR normalized to peak HR after symptom limited cardiopulmonary exercise among control, nonobstructive, and obstructive groups. The change in mean normalized heart rate with time after cessation of exercise is shown, with 95% CIs calculated by the Loess function.

The absolute HRR and HRR normalized to peak HR are shown in Figure 2. The absolute reduction in heart rate at 1 minute was significantly lower in the nonobstructive group (26.4 ± 7.8 beats/min, p = 0.007) and the obstructive group (24.2 ± 9.6 beats/min, p = 0.005), but no difference between the nonobstructive and obstructive groups was observed (p = 1.0). At 2 minutes, the absolute reduction in HR was lower in the nonobstructive (37.8 ± 10.1 beats/min, p <0.001) and obstructive groups (34.5 ± 7.5 beats/min, p <0.001) compared with controls. No difference between the nonobstructive and obstructive groups was observed (p = 1.0). A similar trend was seen at 3 minutes with absolute HR reduction being lower in nonobstructive (43.4 ± 12.8 beats/min, p <0.001) and obstructive groups (36.4 ± 9.7 beats/min, p <0.001) versus controls. No statistical difference was observed between obstructive and nonobstructive patient groups (p = 0.327).

When normalized to the peak heart rate, the percent reduction in peak HR at 1 minute was similar in the control and in the nonobstructive groups (17.9 ± 4.8%, p = 0.458). There was a trend toward a lower HR reduction in the obstructive group compared with the controls (15.6 ± 6.0%, p = 0.08). There was no difference between the patient groups (p = 0.748). At 2 minutes, HRR normalized to peak HR was lower in both nonobstructive (25.4 ± 5.4%, p = 0.015) and obstructive (22.5 ± 4.0%, p = 0.01) groups compared with controls. There was no difference between the disease groups (p = 0.478). At 3 minutes, the normalized recovery in HR was lower in the nonobstructive group (28.9 ± 5.9%, p = 0.008) and the obstructive group (23.8 ± 6.2%, p <0.001) compared with controls. A trend toward a greater reduction in HR in the nonobstructive group was observed compared with the obstructive group (p = 0.09).

When HR recovery was normalized to the difference between HR at peak exercise and rest, the HRR was lower in the obstructive group but did not reach statistical significance at 1 and 2 minutes. At 3 minutes, there was no significant difference between the controls and nonobstructive group (p = 0.726) or between the 2 disease groups (p = 0.146). There was a significant reduction in HRR in the obstructive group compared with the controls (p = 0.019) at 3 minutes.

In model 1 incorporating controls and patients, peak HR (B 0.911, confidence interval [CI] 0.794 to 1.028, p = <0.001) and HC (B 7.226, CI 1.177 to 13.276, p = 0.020) were independent predictors of HR at 1 minute. Peak HR (B 0.803, CI 0.675 to 0.930, p <0.001) and HC (B 11.50, CI 4.883 to 18.117, p = 0.001) were also independent predictors of HR at 2 minutes. Predictors of HR at 3 minutes were peak HR (B 0.584, CI 0.456 to 0.712, p <0.001) and male gender (B 13.608, CI 6.435 to 20.781, p = <0.001).

In model 2 using the HC cohort alone, there was no association between ejection fraction and lateral E/E′ ratio with HR during recovery. Left atrial size was a univariate predictor of HR at 3 minutes but did not remain significant after adjusting for peak HR and gender. MWT was a univariate predictor of HR at 1, 2, and 3 minutes. In multivariable analysis, MWT (B 0.784 beats per min/mm, CI 0.078 to 1.137, p = 0.026) remained an independent predictor for HR at 1 minute after adjusting for peak HR (B 0.608 beats/min/mm, CI 0.078 to 1.137, p = 0.026). Peak LVOT gradient was a univariate predictor of HR at 3 minutes (B 0.154 beats/min/mm Hg, CI 0.010 to 0.299, p = 0.037). In multivariable analysis incorporating peak left ventricular outflow gradient, peak HR, and gender, peak LVOT gradient (B 0.141 beats/min/mm Hg, CI 0.059 to 0.222, p = 0.001) remained an independent predictor of HR at 3 minutes.

Nineteen patients had no sudden cardiac death risk factors, 13 had 1, and 9 had 2 or more. There was no association between the number of risk factors and HRR at 1, 2, and 3 minutes. Five patients had an ABPR to exercise, and 10 patients had NSVT. All indices of HRR were comparable between patients with normal and ABPR to exercise (data not shown). Patients with NSVT demonstrated a smaller reduction in HR at 1 minute when normalized to the difference between HR at peak exercise and rest (p = 0.040). All other indices of HR recovery were comparable in patients with and without NSVT (data not shown).

**Figure 3:**
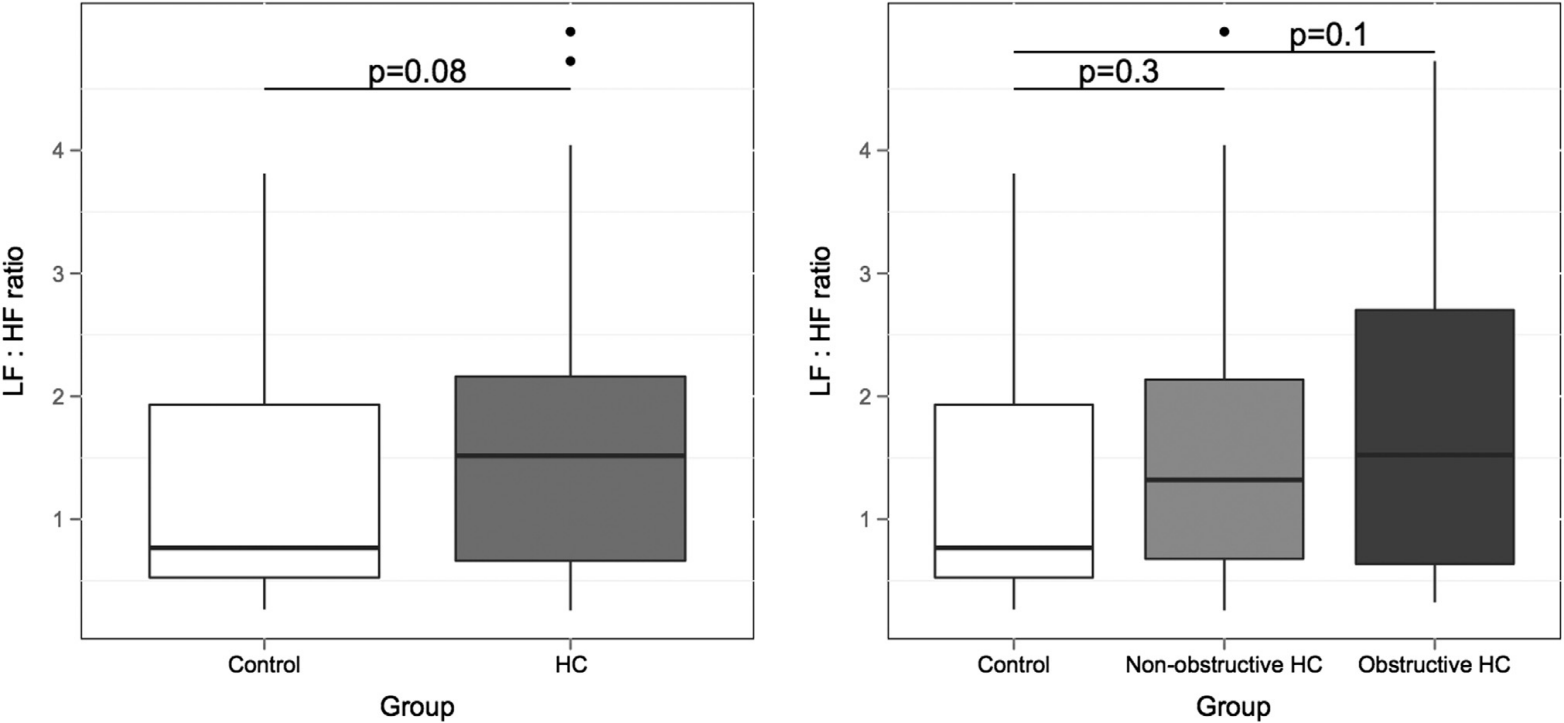
*(A)* Averaged low-frequency to high-frequency (LF:HF) ratio over 3 minutes between control and HC groups. Median and interquartile range. p Values on graph. *(B)* Averaged LF:HF ratio over 3 minutes among control, nonobstructive, and obstructive groups. Median and interquartile range. p Values on graph.

During the 3 minutes of recovery, the low-frequency and high-frequency components were similar between controls and patients (Table 1). The low-frequency to high-frequency ratio was higher in the combined HC group compared the controls with a trend toward significance (p = 0.08; Figure 3). Dichotomization of the HC group by obstruction failed to identify any significant difference among the 3 groups (Table 1 and Figure 3).

## Discussion

In large follow-up studies of healthy adults, impaired HR recovery has been shown to be a predictor of all-cause mortality and, in individuals with cardiac disease, is associated with an increased risk of cardiac and all-cause mortalities.^2–7^ HRR after exercise results from the combined effects of sympathetic withdrawal and parasympathetic reactivation. Although published data are not entirely consistent, studies using parasympathetic and β-adrenergic blockade suggest that the restoration of parasympathetic tone predominates in early HR recovery after exercise in both health and disease.^16^ However, the explanation for the association between abnormal HR recovery and an increased risk of death in various settings is not known.

The results of this study demonstrate that HR recovery is impaired in patients with HC, and that LVOTO is a key determinant of the HRR response. Although there is some variability in the findings of individual studies, most data suggest that autonomic function is abnormal in patients with HC, with the majority showing decreased cardiac parasympathetic activity.^8–10^ The evidence in support of altered sympathetic activity in HC is less consistent with studies showing a reduction in sympathetic tone during activities of daily living and an increase in sympathetic tone in patients with advanced disease.^11,12^ A number of studies have also shown that local cardiac noradrenaline kinetics are altered with reduced synaptic re-uptake, increased myocardial washout rate, and a reduction in β adrenergic receptor density.^17–20^ Few studies have specifically examined autonomic function during exercise in patients with HC, but it is suggested that the common findings of ABPR on exercise^21–24^ may be explained by peripheral vasodilation caused by inappropriate firing of cardiopulmonary mechanoreceptors during central blood volume unloading.^25,26^

We postulate that individuals with HC have a reduction in the vagal influences that determine heart rate kinetics. The association between LVOTO and HRR may be explained by a reduction in arterial baroreceptor stimulation during late systole as a result of altered aortic pressure. Alternatively, it may represent a paradoxical and compensatory response to a reduction in peripheral resistance after activation of cardiopulmonary mechanoreceptors in response to the ventricular stretch and myocardial ischemia generated by LVOTO. Although the data in this study are consistent with abnormal sympathovagal balance after exercise, analysis of the frequency power spectrum only showed a trend toward significance between the control and the combined HC group. Technical limitations have to be considered, including signal noise and artifact that may have been introduced by the recording technique and exaggerated by the short sampling duration. The power spectrum was not assessed during exercise, and environmental factors that can disturb sympathovagal balance and the influence of respiratory rate on the power spectrum were not accounted for. In addition, HRV may not linearly reflect parasympathetic outflow, and saturation of parasympathetic tone may reduce the respiratory variation on heart rate and thus HRV.^27,28^ Physiological and withdrawal effects of cardioactive medications may also persist beyond the time frame chosen in this study, and dietary intake for and the timing of the exercise test were not accounted for. Finally, the use of finger plethysmography may not be accurate at determining the presence of ectopy and atrial arrhythmias. However, we found that finger plethysmography with the use of a splint to maintain the hand in a neutral position was less prone to movement artifact than the surface electrocardiogram during dynamic exercise, and no patient developed atrial or ventricular arrhythmias during exercise.

## Disclosures

The authors have no conflicts of interest to disclose.

## Figures and Tables

**Table 1:** Baseline characteristics and recovery results from the control and disease groups

Variable	Controls (n = 18)	All HC (n = 41)	Nonobstructive (n = 28)	Obstructive (n = 13)
Men	9	36∗	26∗	11∗
Age (yrs)	39.7 ± 10.0	46.7 ± 13.0∗	47.5 ± 13.7	44.9 ± 11.8
Body mass index (kg/m^2^)	24.1 ± 3.0	29.5 ± 5.3∗	29.9 ± 5.4∗	28.9 ± 5.3∗
Mean arterial blood pressure at rest (mm Hg)	90.8 ± 7.5	92.8 ± 12.7	94.3 ± 12.3	89.6 ± 13.4
Systolic blood pressure at rest (mm Hg)	120.3 ± 10.1	124.9 ± 18.5	127.7 ± 16.1	118.8 ± 22.3
Systolic blood pressure at peak (mm Hg)	165.6 ± 21.4	169.5 ± 30.2	171.4 ± 28.8	165.4 ± 33.8
Change in systolic blood pressure (mm Hg)	44.2 ± 8.1	48.2 ± 12.4	50.2 ± 10.7	44.0 ± 15.0
Active smokers	1	4	2	2
NYHA Class 1	18	22	17	5
NYHA Class 2	0	18	10	8
NYHA Class ≥3	0	1	1	0
Resting heart rate (beats/min)	80.5 ± 14.7	76.6 ± 14.51	74.6 ± 15.8	81.1 ± 10.6
Peak heart rate (beats/min)	176.8 ± 18.1	149.8 ± 21.7∗	148.3 ± 24.4∗	153.0 ± 14.7∗
Change in heart rate (beats/min)	96.3 ± 17.2	73.2 ± 21.4∗	73.8 ± 24.2∗	71.9 ± 14.4∗
Maximal oxygen consumption (ml/kg/min)	35.1 ± 8.4	23.7 ± 5.6∗	24.2 ± 6.1∗	22.5 ± 4.4∗
Peak workload achieved (W)	206.1 ± 73.6	143.2 ± 47.5∗	152.3 ± 50.9∗	123.5 ± 32.5∗
Maximal wall thickness (mm)	—	17 ± [14–21]	15.0 ± [13–20.5]	18 ± [16.5–21.5]
Peak left ventricular outflow tract gradient (mm Hg)			6 [4–9.75]	78 [60–100.5]†
Ejection fraction (%)	—	67.0 ± 7.7	66.4 ± 8.6	68.2 ± 5.5
Left atrial diameter (mm)	—	43.7 ± 6.0	44.5 ± 6.3	41.8 ± 5
Mean heart rate at 1 minute (beats/min)	141.4 ± 24.19	124.1 ± 19.3∗	121.9 ± 21.5∗	128.9 ± 13.1
Mean heart rate reduction at 1 minute (beats/min)	35.3 ± 11.0	25.7 ± 8.4∗	26.4 ± 7.8∗	24.2 ± 9.6∗
Reduction of peak heart rate at 1 minute (%)	20.4 ± 6.8	17.2 ± 5.2	17.9 ± 4.8	15.6 ± 6.0
Reduction of absolute change in heart rate during exercise at 1 minute (%)	37.7 ± 13.5	36.3 ± 11.9	37.8 ± 12.3	32.9 ± 10.9
Mean heart rate at 2 minutes (beats/min)	123.2 ± 25.7	113.6 ± 17.1	110.5 ± 18.9	118.5 ± 11.3
Mean heart rate reduction at 2 minutes (beats/min)	53.6 ± 13.2	36.8 ± 9.4∗	37.8 ± 10.1∗	34.5 ± 7.5∗
Reduction of peak heart rate at 2 minutes (%)	30.7 ± 7.8	24.5 ± 5.2∗	25.4 ± 5.4∗	22.5 ± 4.0∗
Reduction of absolute change in heart rate during exercise at 2 minutes (%)	56.6 ± 13.9	51.7 ± 10.9	53.2 ± 11.9	48.4 ± 8.0
Mean heart rate at 3 minutes (beats/min)	114.7 ± 24.1	108.6 ± 16.8	104.9 ± 16.6	116.6 ± 14.7
Mean heart rate reduction at 3 minutes (beats/min)	62.1 ± 14.5	41.2 ± 12.2∗	43.4 ± 12.8∗	36.4 ± 9.7∗
Reduction of peak heart rate at 3 minutes (%)	35.5 ± 8.6	27.3 ± 6.4∗	28.9 ± 5.9∗	23.8 ± 6.2∗
Reduction of absolute change in heart rate during exercise at 3 minutes (%)	65.7 ± 15.8	57.8 ± 13.5	60.7 ± 12.4	51.4 ± 13.9∗
Normalized low frequency spectral power (n.u)	0.48 ± 0.19	0.56 ± 0.18	0.55 ± 0.18	0.56 ± 0.19
Normalized high frequency spectral power (n.u)	0.51 ± 0.19	0.44 ± 0.18	0.45 ± 0.18	0.44 ± 0.19
Low:high frequency ratio	0.77 [0.53–1.93]	1.52 [0.66–2.16]	1.32 [0.68–2.14]	1.52 [0.64–2.70]

Normally distributed continuous data are expressed as mean ± SD and as median and interquartile range for non-normally distributed data. NYHA = New York Heart Association.

∗ p <0.05 versus controls.

† p <0.05 versus nonobstructive.
